# Adjunctive Autonomic Regulation Practices in Pelvic Floor Rehabilitation: A Retrospective Comparative Cohort Study

**DOI:** 10.3390/jcm15218375

**Published:** 2026-10-27

**Authors:** Özdal Ersoy, Mervenur Akçay, Yasemin Temel Pekmez, Fevzi Kılıçkan

**Affiliations:** 1 Department of Internal Medicine and Gastroenterology, Medical Faculty, Istanbul Arel University, 34010 Istanbul, Türkiye; 2 Pelvic Floor Physiotherapy Unit, Bahcelievler Memorial Hospital, 34180 Istanbul, Türkiye; mervenur.tapan@memorial.com.tr; 3 Department of Occupational Therapy, Faculty of Health Sciences, Istanbul Atlas University, 34408 Istanbul, Türkiye; yaseminecemtemel@hotmail.com; 4 Institute of Graduate Education, Istinye University, 34396 Istanbul, Türkiye; 5 Department of Public Health, Cerrahpasa Faculty of Medicine, Istanbul University-Cerrahpasa, 34098 Istanbul, Türkiye; duhankilickan@gmail.com

**Keywords:** pelvic floor dysfunction, pelvic floor physiotherapy, biofeedback, autonomic regulation, vagus nerve, MYMOP2, constipation, pelvic pain

## Abstract

**Background/Objectives:** Pelvic floor physiotherapy (PFPT) and biofeedback are established conservative treatments for pelvic floor dysfunctions, but clinical response is heterogeneous. We explored whether exposure to a structured multicomponent set of non-device home practices, described here as adjunctive autonomic regulation practices, was associated with patient-reported improvement when used alongside standard PFPT/biofeedback. The study was exploratory and was not designed to establish an autonomic mechanism or causal treatment effect. **Methods:** This retrospective, observational, nonconcurrent comparative cohort study included adults treated in the same tertiary pelvic floor rehabilitation unit during two distinct periods. The adjunct-exposed cohort (Group A; September 2022–March 2023) received PFPT/biofeedback plus a structured multicomponent home program; the historical comparator (Group B; January–June 2019) received PFPT/biofeedback without the adjunct. Both programs included diaphragmatic breathing; in the standard program, no fixed breathing rate or duration was prescribed, whereas the adjunct included slow-paced breathing at approximately 6 breaths/min for 5 min three times daily. The primary outcome was the proportion of patients classified as responders according to improvement in the MYMOP2 Profile Score from baseline to approximately 2 months. The historical dataset operationalized clinically meaningful response as >1.0-point improvement. Between-group analyses were unadjusted. **Results:** A total of 111 patients were analyzed (Group A, *n* = 35; Group B, *n* = 76). MYMOP2 response occurred in 31/35 patients (88.6%) in Group A and 42/76 patients (55.3%) in Group B (Pearson chi-square: chi2(1) = 11.81, *p* < 0.001). The unadjusted risk ratio (RR) was 1.60 (95% CI 1.27–2.03), and the unadjusted absolute difference was 33.3 percentage points (95% CI 17.9–48.7). Using recently proposed physiotherapy-specific RR reference thresholds at a baseline risk near 0.5, the numerical RR lies in the upper medium range and close to the proposed large-effect threshold; this describes magnitude only and does not imply a causal treatment effect. **Conclusions:** Patients exposed to the adjunctive multicomponent program had a higher unadjusted MYMOP2 responder rate than patients in a nonconcurrent historical standard-rehabilitation cohort. Because the cohorts were separated in time, baseline characterization was incomplete, follow-up availability may have introduced selection bias, historical patient-level MYMOP2 scores are no longer accessible, and the intervention was multicomponent, the findings should be interpreted as exploratory and hypothesis-generating. Prospective studies with concurrent controls, standardized treatment delivery, objective pelvic floor outcomes, and prospectively measured adherence are required.

## 1. Introduction

Pelvic floor dysfunctions comprise a heterogeneous group of conditions involving impaired coordination, strength, relaxation, endurance, or neuromuscular control of the pelvic floor. Clinical presentations include obstructed defecation and chronic constipation, fecal or urinary incontinence, pelvic pain, and related anorectal or pelvic symptoms [1,2,3,4,5,6]. Pelvic floor rehabilitation commonly combines education, toileting and defecation training, pelvic floor muscle training or down-training, diaphragmatic breathing, core coordination, and visual, tactile, or electromyographic biofeedback. The diaphragm, abdominal wall, and pelvic floor also function as an integrated pressure-regulation and core system, although the evidence supporting specific breathing-based rehabilitation techniques remains heterogeneous [7].

Stress, heightened arousal, altered breathing patterns, and symptom-related muscle guarding may coexist with pelvic floor symptoms and may interfere with relaxation, coordination, and symptom self-management. Parasympathetic and vagal pathways are relevant to gastrointestinal function, visceral processing, and inflammatory signaling [8,9], while slow breathing can influence cardiorespiratory autonomic indices [10,11]. These observations provide biological plausibility for studying relaxation-oriented behavioral practices alongside pelvic floor rehabilitation, but they do not establish that pelvic floor dysfunction is primarily an autonomic disorder or that non-device practices are equivalent to electrical vagus nerve stimulation.

The adjunct evaluated in this study was deliberately multicomponent. It included structured slow-paced diaphragmatic breathing together with cold-water facial stimulation, vocal practices, meditation/relaxation, and general behavioral recommendations. The individual components have been associated with physiological or psychophysiological responses in other populations [10,11,12,13,14,15,16,17,18], but the evidence is indirect and does not support a single unified mechanism. Moreover, standard pelvic floor rehabilitation itself included diaphragmatic breathing; the treatment contrast therefore concerns a broader structured home program and a more specific breathing dose rather than breathing versus no breathing.

To our knowledge, this combination of non-device practices has not been evaluated as a structured adjunct to PFPT/biofeedback in a clinical pelvic floor population. The objective of this retrospective, nonconcurrent comparative cohort study was to explore whether exposure to the adjunctive multicomponent program was associated with a higher proportion of clinically meaningful MYMOP2 improvement at approximately 2 months than a historical cohort receiving standard rehabilitation alone. The analysis was intended to identify a hypothesis-generating clinical signal, not to establish causality or an autonomic/vagal mechanism.

## 2. Materials and Methods

### 2.1. Study Design and Setting

This retrospective, observational, nonconcurrent comparative cohort study was conducted in a tertiary pelvic floor rehabilitation unit. The study was approved by the Acibadem University School of Medicine Ethics Committee, Istanbul Türkiye (No. 2023-10/330; June 2023). The written study protocol submitted before the retrospective comparative analysis described comparisons of patients treated during two clinical periods and specified MYMOP2 assessment before treatment and at approximately 2 months. The study protocol did not prospectively register the >1.0-point responder threshold. Reporting follows the STROBE framework, provided as Supplementary File S1 [19].

The study used previously collected clinical data. The ethics submission included a study-specific informed consent form describing the retrospective use and comparison of previous clinical data. Participants provided written informed consent for participation and for use of their previous clinical data. No waiver of study-specific consent is claimed.

### 2.2. Participants

Adults (≥18 years) treated for clinically diagnosed pelvic floor dysfunction and enrolled in the pelvic floor rehabilitation program were eligible for the retrospective analysis. Pelvic floor dysfunction was established clinically according to the presenting anorectal/pelvic symptoms and routine specialist assessment, with additional investigations used when clinically indicated. Inclusion in the analytic cohort required (1) a baseline MYMOP2 assessment before treatment, (2) a follow-up MYMOP2 assessment at approximately 2 months, and (3) completion of the relevant rehabilitation pathway. Patients with missing baseline or follow-up MYMOP2 data or a major concurrent intervention expected to substantially alter the primary symptoms were excluded. Because complete screening logs for both historical periods are no longer available, the total number of otherwise eligible patients excluded because of absent follow-up MYMOP2 data cannot be reliably reconstructed; this creates potential selection bias. No formal sample-size calculation was performed because all available eligible records from the defined periods were analyzed.

### 2.3. Cohort Definition

Group A (core rehabilitation + adjunct): 35 patients treated between September 2022 and March 2023 who received PFPT/biofeedback plus the adjunctive multicomponent home practices.

Group B (historical core rehabilitation): 76 patients treated between January and June 2019 who received PFPT/biofeedback without the adjunct. This cohort was independent of the previously published Turkish MYMOP2 validation cohort. The validation study comprised a separate prospective sample of 50 patients with anorectal disorders and is cited here solely to document the validity and reliability of the Turkish MYMOP2 instrument [20]. None of those validation participants were included as the historical comparator in the present study. Because Groups A and B were treated in different calendar periods, exchangeability cannot be assumed; changes in referral patterns, therapist experience, treatment delivery, documentation, equipment, or organization of care may have contributed to between-group differences.

### 2.4. Core Pelvic Floor Physiotherapy and Biofeedback Program

Both cohorts received individualized PFPT/biofeedback delivered by pelvic floor physiotherapists. The routine program included education regarding bowel and pelvic floor function; dietary fiber and fluid advice when appropriate; toileting posture and defecation training; abdominal massage when indicated; pelvic floor and core exercises emphasizing relaxation, coordination, or strengthening according to clinical findings; and EMG-assisted or non-instrumented biofeedback as appropriate. Diaphragmatic breathing was part of standard rehabilitation, but the historical protocol did not prescribe a fixed breathing rate or standardized duration. Treatment content was individualized, and complete patient-level records of the number of sessions and the proportion receiving each optional component are not available consistently across both periods. Therefore, identical treatment exposure across cohorts cannot be verified.

### 2.5. Adjunctive Autonomic Regulation Practices

Group A additionally received a structured set of non-device home practices intended to support relaxation, behavioral self-regulation, and symptom management shown in Table 1. The contemporaneous study protocol included slow/deep diaphragmatic breathing at approximately 6 breaths/min for 5 min three times daily, with expiration longer than inspiration; gargling for approximately 30–60 s; humming or singing; morning and evening cold-water facial washing; meditation/relaxation practice; and additional behavioral/lifestyle suggestions. The program was pragmatic and multicomponent rather than a standardized device-based vagus nerve stimulation intervention. Exact water temperature was not standardized or recorded, and some lifestyle elements were not delivered with objectively monitored dose or fidelity. The term ‘autonomic regulation practices’ is therefore used descriptively and should not be interpreted as evidence that the clinical association was mediated by autonomic or vagal mechanisms.

### 2.6. Outcome Measure

The primary outcome was the proportion of patients classified as having clinically meaningful improvement in the MYMOP2 Profile Score from baseline to approximately 2 months. MYMOP2 is a patient-generated outcome measure in which patients identify symptoms of greatest importance, an activity affected by the problem, and general wellbeing; lower scores indicate improvement [21,22]. The Turkish version had previously been validated in a separate prospective cohort of 50 patients with anorectal disorders [20]. A 1.0-point MYMOP change has been used in the literature as a minimal clinically important difference [23]. In the historical study dataset used for the present comparison, response had been operationalized as >1.0-point improvement in the MYMOP2 Profile Score. This threshold was not derived from the present between-group results to maximize statistical significance; however, it was not explicitly prospectively registered in the 2023 study protocol, and we therefore do not describe the dichotomous responder definition as prespecified. For Group B, the retained responder classification was available, but the original patient-level baseline and follow-up MYMOP2 scores are no longer accessible. Consequently, continuous change, baseline severity, and independent reconstruction of historical responder status cannot be performed.

### 2.7. Statistical Analysis

Categorical variables were summarized as counts and percentages. The primary unadjusted comparison of responder proportions used the Pearson chi-square test. Effect estimates are reported as an unadjusted RR with 95% CI and an unadjusted absolute difference in responder proportions with 95% CI. Because the historical cohort lacked sufficiently complete individual-level baseline covariates, a reliable multivariable adjustment was not possible. Symptom-category analyses were exploratory and descriptive only, with no inferential testing because of small cell sizes. For contextual interpretation of the numerical RR, we used recently proposed physiotherapy-specific reference thresholds that depend on baseline risk [24]. At p0 = 0.5, the proposed RR thresholds are 1.09, 1.35, and 1.62 for small, medium, and large effects, respectively; these labels describe numerical magnitude and do not convert an unadjusted historical comparison into a causal effect estimate. Analyses were performed using IBM SPSS Statistics version 25.0. No missing outcome data were imputed.

### 2.8. Sex and Gender Considerations

Sex distribution was reported for each cohort. Gender identity was not routinely captured in the retrospective clinical records and therefore could not be analyzed.

## 3. Results

### 3.1. Participant Characteristics

A total of 111 patients met the analytic inclusion criteria: 35 in Group A and 76 in Group B. Group A included 7 men (20.0%) and 28 women (80.0%); Group B included 29 men (38.2%) and 47 women (61.8%). The sex distributions differed numerically, although the unadjusted comparison did not reach conventional statistical significance (Pearson chi-square *p* = 0.058). This does not establish baseline comparability, and sex remains a potential confounder. Primary symptom categories are summarized in Table 2. Other potentially important baseline variables, including age, baseline MYMOP2 severity, symptom duration, comorbidity burden, medication use, previous treatments, and psychological distress, were not consistently recoverable across both cohorts and therefore could not be used for adjusted analyses.

### 3.2. Primary Outcome

MYMOP2 response (>1.0-point improvement in the Profile Score) occurred in 31 of 35 patients (88.6%) in Group A and 42 of 76 patients (55.3%) in Group B. The unadjusted absolute difference was 33.3 percentage points (95% CI 17.9–48.7), and the unadjusted RR was 1.60 (95% CI 1.27–2.03); Pearson chi-square chi2(1) = 11.81, *p* < 0.001. With a historical responder proportion of 55.3%, the p0 = 0.5 RR thresholds proposed for physiotherapy provide the closest reference scenario: RR 1.60 lies in the upper medium range and close to the proposed large threshold of 1.62 [24]. This classification concerns numerical magnitude only. Because the cohorts were nonconcurrent and incompletely characterized at baseline, the estimate should not be interpreted as a causal treatment effect.

### 3.3. Symptom-Category Patterns

Descriptive response patterns by primary symptom category are shown in Table 3. Group A response occurred in 5/5 patients with pelvic pain, 19/21 with constipation/obstructed defecation, 4/6 with incontinence, and 3/3 in the other category. Corresponding Group B counts were 5/15, 27/42, 7/11, and 3/8. These subgroup results are based on small and, in some categories, extremely small denominators; they are statistically unstable, were not tested inferentially, and should not be interpreted as evidence of differential effectiveness by symptom phenotype.

### 3.4. Documented Adverse Events

No serious adverse events attributed to the adjunctive practices were documented in the available clinical records. Minor tolerance issues could have been under-recorded because adverse events were not collected prospectively using a standardized safety instrument. The absence of documented events should therefore not be interpreted as proof of absence of minor adverse effects.

## 4. Discussion

In this retrospective, nonconcurrent comparative cohort study, patients exposed to standard PFPT/biofeedback plus a structured multicomponent home program had a higher unadjusted MYMOP2 responder rate at approximately 2 months than patients in a historical standard-rehabilitation cohort (88.6% vs. 55.3%; RR 1.60, 95% CI 1.27–2.03; absolute difference 33.3 percentage points, 95% CI 17.9–48.7). The numerical RR lies in the upper medium range and close to the proposed large-effect threshold for a baseline risk near 0.5 [24]. This effect-size description does not establish causality. The nonconcurrent design, incomplete baseline characterization, and inability to adjust for important confounders mean that the observed association may reflect treatment exposure, temporal changes, baseline differences, or combinations of these factors.

The clinical rationale for examining the adjunct was that breathing strategy, arousal, stress, and pelvic floor muscle guarding may interact with pelvic floor relaxation and coordination in some patients. Slow breathing can influence cardiorespiratory autonomic measures [10,11], and the diaphragm, abdominal wall, and pelvic floor participate in coordinated pressure regulation [7]. However, the present study measured neither autonomic physiology nor objective pelvic floor neuromuscular function. These mechanisms therefore remain hypotheses rather than demonstrated mediators of the observed patient-reported response.

The adjunct was also multicomponent. In addition to structured slow-paced breathing, it included cold-water facial stimulation, vocal practices, meditation/relaxation, and behavioral/lifestyle recommendations. Physiological studies of humming or cold-face stimulation [14,15,16,17] and studies of meditation-related autonomic measures [18] provide component-level context, but they cannot identify the active element in this clinical program. Moreover, standard rehabilitation already included diaphragmatic breathing without a fixed rate or duration. The observed association therefore cannot be attributed specifically to paced breathing, vagal activity, autonomic regulation, or any single component.

Patient-reported outcomes may also be influenced by nonspecific factors. Participants in the adjunct period were aware that they had received additional instructions, and the program may have increased therapeutic contact, engagement, expectations, or self-management attention. Sleep-related advice, physical activity, relaxation, and behavioral engagement could also influence symptoms independently of any proposed autonomic pathway. These possibilities are particularly relevant because the study was unblinded and MYMOP2 was the primary outcome.

Accordingly, the present findings are best viewed as a hypothesis-generating clinical signal. They support prospective evaluation of the multicomponent program but do not support a clinical recommendation for ‘vagal stimulation’ or demonstrate that autonomic modulation explains the observed difference. Future work should separate the effects of additional treatment attention from specific intervention components and, where mechanistic claims are intended, incorporate objective physiological measures.

This study has several important limitations. First, its retrospective design precludes causal inference. Second, the cohorts were nonconcurrent and separated by several years, creating substantial potential for temporal confounding from changes in referral patterns, therapist experience, treatment delivery, documentation, equipment, or organization of care. Third, baseline characterization was limited; age, baseline MYMOP2 severity, symptom duration, comorbidities, medications, previous treatments, and psychological distress were not consistently recoverable, and the observed sex imbalance may have also confounded the comparison. Fourth, requiring both baseline and follow-up MYMOP2 data may have introduced selection bias, and complete screening/loss-to-follow-up counts could not be reconstructed. Fifth, the original patient-level baseline and follow-up MYMOP2 scores for the historical cohort are no longer accessible; therefore, baseline severity, continuous change distributions, regression to the mean, and historical responder status cannot be independently reconstructed. Sixth, adherence was based on routine self-report rather than prospective fidelity monitoring. Seventh, adverse events were identified retrospectively rather than through systematic safety surveillance. Eighth, the intervention was multicomponent, preventing attribution to any individual practice or to autonomic regulation. Ninth, the primary outcome was patient-reported and unblinded, leaving expectation and additional-attention effects possible. Finally, no objective pelvic floor outcomes (for example, measures of activation, relaxation, coordination, pressure, or anorectal physiology) and no autonomic markers such as heart-rate variability were included.

Prospective studies should use concurrent control groups, standardized therapist delivery, transparent participant flow, comprehensive baseline characterization, prospectively measured adherence and treatment fidelity, continuous as well as responder-based patient-reported outcomes, and objective pelvic floor/anorectal outcomes where appropriate. If an autonomic mechanism is hypothesized, physiological measures such as heart-rate variability should be incorporated. Randomized or carefully matched prospective designs and factorial/dismantling approaches would help distinguish the effects of additional treatment attention, structured slow breathing, relaxation, lifestyle advice, and other components.

## 5. Conclusions

In this retrospective, nonconcurrent comparison, patients exposed to PFPT/biofeedback plus a structured multicomponent adjunct had a higher unadjusted MYMOP2 responder rate than patients in a historical standard-rehabilitation cohort. The result should be interpreted as an exploratory, hypothesis-generating association rather than evidence that the adjunct caused the improvement or that an autonomic/vagal mechanism was responsible. Prospective concurrent controlled studies with standardized treatment delivery, adequate baseline adjustment, objective pelvic floor outcomes, and prospectively assessed adherence are required before clinical effectiveness or mechanism can be established.

## Figures and Tables

**Table 1:** Components of the adjunctive autonomic regulation practices (Group A).

Prescribed Practice	Protocol Description	Intended/Hypothesized Rationale
Structured slow diaphragmatic breathing	Deep/slow breathing at approximately 6 breaths/min for 5 min, three times daily; expiration longer than inspiration	Relaxation and breathing/pelvic-floor coordination; hypothesized autonomic support
Cold-water facial stimulation	Cold-water facial washing in the morning and evening; exact water temperature was not standardized or recorded	Sensory stimulation; hypothesized autonomic engagement
Vocal/oropharyngeal practices	Gargling for approximately 30–60 s; humming or singing encouraged during the day	Respiratory-vocal engagement and relaxation
Meditation/relaxation	Meditation/relaxation practice was encouraged as part of the home program; exact individual dose was not objectively monitored	Reduction in arousal and muscle guarding
Behavioral/lifestyle reinforcement	General recommendations included social engagement, positive/pleasant sensory experiences, time in nature or calming activities, and related self-regulation practices	General behavioral engagement and stress-management support

Adherence information was obtained during routine clinical follow-up from patient self-report and clinical documentation. No prospective daily diary, electronic monitoring, or objective fidelity measure was used. Accordingly, absence of documented non-adherence cannot be equated with verified completion of every component, and the actual frequency, duration, and fidelity of individual home practices cannot be reconstructed reliably.

**Table 2:** Baseline primary symptom categories.

Primary Symptom	Group A (*n* = 35)	Group B (*n* = 76)
Constipation/obstructed defecation	21 (60.0%)	42 (55.3%)
Pelvic pain	5 (14.3%)	15 (19.7%)
Incontinence	6 (17.1%)	11 (14.5%)
Other *	3 (8.6%)	8 (10.5%)

* Other included irritable bowel syndrome symptoms and rectal prolapse symptoms.

**Table 3:** Clinically meaningful MYMOP2 response by primary symptom category.

Primary Symptom	Group A, Responders/*n*	Group A, %	Group B, Responders/*n*	Group B, %
Pelvic pain	5/5	100.0	5/15	33.3
Constipation/obstructed defecation	19/21	90.5	27/42	64.3
Incontinence	4/6	66.7	7/11	63.6
Other *	3/3	100.0	3/8	37.5

* Other included irritable bowel syndrome symptoms and rectal prolapse symptoms. Subgroup results are descriptive only.

## Data Availability

Aggregate data supporting the reported analyses are available from the corresponding author on reasonable request, subject to applicable ethical and privacy restrictions. The original patient-level baseline and follow-up MYMOP2 scores for the historical 2019 cohort are no longer accessible; retained responder classifications and aggregate results were used in the present analysis. Consequently, the historical continuous change scores and responder classifications cannot be independently reconstructed from raw MYMOP2 forms.

## Supplementary Materials

The following supporting information can be downloaded at: https://www.mdpi.com/article/10.3390/jcm15218375/s1, Supplementary File S1, completed STROBE checklist for cohort studies.
