# Supplementary material for: Adjunctive Autonomic Regulation Practices in Pelvic Floor Rehabilitation: A Retrospective Comparative Cohort Study
Source: J Clin Med. 2026 Oct 27;15(21):8375. doi: 10.3390/jcm15218375 (PMC13763776; doi:10.3390/jcm15218375)
Supplement: Supplementary file 1 [file jcm-15-08375-s001.zip › jcm-4599111-supplementary.pdf]

Supplementary File S1

STROBE Statement - Checklist of Items for Cohort Studies

Manuscript: Adjunctive Autonomic Regulation Practices in Pelvic Floor Rehabilitation: A Retrospective Comparative Cohort Study

Updated to correspond to the Reviewer 3 Round 2 revised manuscript. Items not applicable or not performed are retained transparently rather than inferred or reconstructed from unavailable historical data.

Section	Item	STROBE recommendation	Where reported	Completion note
Title and abstract	1a	Indicate the study design with a commonly used term in the title or abstract.	Title; Abstract - Methods	Reported as a retrospective, observational, nonconcurrent comparative cohort study.
Title and abstract	1b	Provide an informative and balanced summary of what was done and what was found.	Abstract	Structured Background/Objectives, Methods, Results, and Conclusions report unadjusted effect estimates and explicitly note nonconcurrent cohorts, incomplete baseline characterization, unverifiable rehabilitation-dose equivalence, selection bias, unavailable historical MYMOP2 raw scores, and the multicomponent intervention.
Background/rationale	2	Explain the scientific background and rationale for the investigation.	Introduction, paragraphs 1-3	Pelvic floor rehabilitation, breathing/arousal rationale, and component-level autonomic literature are described without asserting a demonstrated autonomic mechanism.
Objectives	3	State specific objectives, including any prespecified hypotheses.	Introduction, final paragraph	Objective is to explore whether adjunct exposure was associated with a higher MYMOP2 responder proportion. Responder is defined as >1.0-point improvement; causal and autonomic/vagal inference is explicitly excluded.
Study design	4	Present key elements of study design early in the paper.	Methods 2.1	Retrospective, observational, nonconcurrent comparative cohort design is stated.
Setting	5	Describe setting, locations, and relevant dates, including recruitment/exposure/follow-up/data collection periods.	Methods 2.1 and 2.3	Tertiary pelvic floor rehabilitation unit; Group A September 2022-March 2023; Group B January-June 2019; outcome at approximately 2 months.
Participants	6a	Give eligibility criteria and sources/methods of participant selection; describe follow-up.	Methods 2.2-2.3	Adult eligibility, MYMOP2 requirements, exclusion criteria, cohort sources, and approximately 2-month follow-up are reported. Pelvic floor dysfunction was diagnosed clinically by the treating specialist team; additional investigations were performed when clinically indicated. Identical diagnostic assessment across periods cannot be verified.
Participants	6b	For matched studies, give matching criteria and numbers of exposed and unexposed.	Not applicable	The cohorts were not matched.
Variables	7	Clearly define outcomes, exposures, predictors, potential confounders/effect modifiers, and diagnostic criteria if applicable.	Methods 2.2-2.7	Exposure is the adjunctive multicomponent home program; primary outcome is responder status defined as >1.0-point MYMOP2 improvement. Diagnostic ascertainment, sex, calendar period, baseline severity, rehabilitation exposure, and other incompletely available covariates are recognized as potential sources of confounding or heterogeneity.
Data sources/measurement	8	For each variable, give sources and assessment methods; describe comparability between groups.	Methods 2.2-2.7; Table 1	Routine clinical records and MYMOP2 assessments were used. The same responder rule was applied to both cohorts. Structured prescribed practices, encouraged meditation/relaxation, and general lifestyle recommendations are distinguished. Identical diagnostic assessment and equivalent rehabilitation dose across periods cannot be verified.
Bias	9	Describe efforts to address potential sources of bias.	Methods 2.2-2.7; Discussion limitations	Common clinical setting and outcome definition are described; subgroup inference was avoided. Selection bias, nonconcurrent cohorts, diagnostic/temporal/therapist differences, unverifiable rehabilitation dose, sex imbalance, adherence limitations, unavailable historical MYMOP2 raw scores, and residual confounding are explicitly discussed.
Study size	10	Explain how the study size was arrived at.	Methods 2.2	All available eligible retrospective records from the defined periods were analyzed; no formal sample-size calculation was performed.
Quantitative variables	11	Explain how quantitative variables were handled; describe groupings and why.	Methods 2.6-2.7	MYMOP2 change was dichotomized using the historical operational definition of >1.0-point improvement. This threshold is not presented as a population-specific MCID and was not prospectively registered for the present comparison.
Statistical methods	12a	Describe statistical methods, including methods used to control for confounding.	Methods 2.7	Pearson chi-square, unadjusted RR with 95% CI, and unadjusted absolute responder-rate difference with 95% CI are reported. Reliable multivariable adjustment was not possible because sufficiently complete linked historical covariate/outcome data were unavailable.

Section	Item	STROBE recommendation	Where reported	Completion note
Statistical methods	12b	Describe methods used to examine subgroups and interactions.	Methods 2.7; Results 3.3	Symptom-category analyses were exploratory and descriptive; no inferential subgroup testing was performed because of small cell sizes.
Statistical methods	12c	Explain how missing data were addressed.	Methods 2.2 and 2.7	Patients missing baseline or follow-up MYMOP2 were excluded by eligibility criteria; no missing outcome data were imputed.
Statistical methods	12d	If applicable, explain how loss to follow-up was addressed.	Methods 2.2	Follow-up MYMOP2 at approximately 2 months was required for inclusion. Complete screening/loss-to-follow-up counts could not be reconstructed; potential selection bias is explicitly acknowledged.
Statistical methods	12e	Describe any sensitivity analyses.	Methods 2.7; Results 3.1	A sex-adjusted sensitivity analysis could not be performed because individual-level linkage between sex and responder status was not retained in a form permitting valid re-analysis. Historical patient-level MYMOP2 scores and sufficiently complete shared baseline covariates were unavailable.
Participants	13a	Report numbers at each stage of study.	Results 3.1	111 patients met analytic inclusion criteria and were analyzed: 35 in Group A and 76 in Group B.
Participants	13b	Give reasons for non-participation at each stage.	Methods 2.2; Discussion limitations	Exclusion criteria are stated. Counts screened/excluded by reason and complete loss-to-follow-up counts were not available and cannot be reconstructed reliably.
Participants	13c	Consider use of a flow diagram.	Not included	A flow diagram was not included because screening-stage counts were unavailable; analyzed cohort numbers are reported directly.
Descriptive data	14a	Give participant characteristics and information on exposures and potential confounders.	Results 3.1; Table 2	Sex distribution and primary symptom categories are reported separately by cohort. Other important baseline variables were not consistently recoverable. Sex imbalance is explicitly identified as a potential confounder.
Descriptive data	14b	Indicate number of participants with missing data for each variable of interest.	Methods 2.2; Results 3.1	Complete baseline/follow-up MYMOP2 was required for inclusion. Missingness for other potential baseline covariates could not be quantified consistently across historical cohorts.
Descriptive data	14c	Summarize follow-up time.	Methods 2.2 and 2.6	Outcome assessment occurred approximately 2 months after treatment initiation; exact patient-level follow-up duration was not available for summary.
Outcome data	15	Report numbers of outcome events or summary measures over time.	Results 3.2-3.3; Table 3	Responders: Group A 31/35 (88.6%); Group B 42/76 (55.3%); symptom-category responder counts are also reported.
Main results	16a	Give unadjusted and, if applicable, adjusted estimates with precision; state confounders adjusted for.	Results 3.2	Unadjusted RR 1.60 (95% CI 1.27-2.03); absolute difference 33.3 percentage points (95% CI 17.9-48.7). No adjusted estimate was possible.
Main results	16b	Report category boundaries when continuous variables were categorized.	Methods 2.6; Results 3.2	Responder was defined as >1.0-point improvement in the MYMOP2 Profile Score. The threshold is described as an operational responder definition, not as an established pelvic-floor population-specific MCID.
Main results	16c	If relevant, translate relative risk into absolute risk for a meaningful time period.	Results 3.2	Absolute responder-rate difference at approximately 2 months is reported as 33.3 percentage points (95% CI 17.9-48.7).
Other analyses	17	Report other analyses, e.g., subgroup/interactions/sensitivity analyses.	Results 3.3; Table 3; Methods 2.7	Descriptive symptom-category analyses are reported. No interaction testing was performed. Sex-adjusted sensitivity analysis was not possible because valid linked historical sex/outcome data were unavailable.
Key results	18	Summarize key results with reference to study objectives.	Discussion, opening paragraph	Higher unadjusted MYMOP2 responder proportion with adjunct exposure is summarized with explicit caution against causal interpretation and acknowledgement that rehabilitation-dose equivalence could not be verified.
Limitations	19	Discuss limitations, including direction/magnitude of potential bias or imprecision.	Discussion, limitations paragraph	Retrospective/nonconcurrent design; temporal, diagnostic, therapist and rehabilitation-exposure differences; incomplete baseline covariates; sex imbalance without valid adjusted analysis; selection bias from follow-up requirement; unavailable historical raw MYMOP2 scores and inability to re-verify individual historical responder classifications; self-reported adherence; retrospective safety ascertainment; multicomponent intervention; unblinded patient-reported outcome; and lack of objective pelvic-floor/autonomic measures are discussed.
Interpretation	20	Give a cautious overall interpretation considering objectives, limitations, multiplicity, similar studies and other evidence.	Discussion paragraphs 1-5; Conclusions	Findings are framed as an exploratory, hypothesis-generating unadjusted association. The manuscript does not attribute the observed difference to paced breathing, vagal activity, autonomic regulation, or any single component.

Section	Item	STROBE recommendation	Where reported	Completion note
Generalisability	21	Discuss generalisability (external validity).	Discussion limitations/future directions; Conclusions	Single tertiary pelvic floor unit, heterogeneous symptoms, nonconcurrent historical cohorts, incomplete baseline characterization, unverifiable rehabilitation-dose equivalence, and pragmatic multicomponent delivery limit generalisability; prospective concurrent studies are recommended.
Funding	22	Give source of funding and role of funders.	Funding statement	The research received no external funding.

Note: The STROBE checklist is a reporting guide, not a study-quality score. Items marked not applicable or not performed are retained transparently rather than inferred or reconstructed from unavailable data.

Reference: von Elm E, Altman DG, Egger M, et al. The Strengthening the Reporting of Observational Studies in Epidemiology (STROBE) Statement: Guidelines for reporting observational studies. PLoS Med. 2007;4:e296.
